# Residential greenness, activities of daily living, and instrumental activities of daily living: A longitudinal cohort study of older adults in China

**DOI:** 10.1097/EE9.0000000000000065

**Published:** 2019-08-30

**Authors:** Anna Zhu, Lijing L. Yan, Chih-Da Wu, Peter James, Yi Zeng, John S. Ji

**Affiliations:** a Environmental Research Center, Duke Kunshan University, Kunshan, China; b Global Health Research Center, Duke Kunshan University, Kunshan, China; c Department of Geomatics, National Cheng Kung University, Tainan, Taiwan; d Department of Population Medicine, Harvard Medical School and Harvard Pilgrim Health Care Institute, Boston, Massachusetts; e Center for the Study of Aging and Human Development, Duke Medical School, Durham, North Carolina; f Center for Healthy Aging and Development Studies, National School of Development and Raissun Institute for Advanced Studies, Peking University, Beijing, China; g Nicholas School of the Environment, Duke University, Durham, North Carolina.

**Keywords:** Activities of Daily Living, Healthy Longevity, Instrumental Activities of Daily Living, Residential Greenness

## Abstract

**Background:**

We aimed to explore whether higher levels of residential greenness were related to lower odds of disabilities in activities of daily living (ADL) and instrumental activities of daily living (IADL).

**Methods:**

We included older adults 65 years of age or older from the Chinese Longitudinal Healthy Longevity Survey. Our exposure was Normalized Difference Vegetation Index in 500 m radius around residence. Our outcome was ADL and IADL. We used binary logistic regression and mixed-effects logistic regression to estimate the odds of ADL and IADL disabilities.

**Results:**

A total of 36,803 and 32,316 participants were included for the analysis of ADL and IADL, with 71.6% free of ADL disability and 47.3% free of IADL disability. In the logistic regression model, compared with the participants living in the lowest quartile of residential greenness, those in the highest quartile had a 28% (odds ratio [OR] = 0.72; 95% confidence interval [CI] = 0.65, 0.79) lower odds of ADL disability and a 14% (OR = 0.86; 95% CI = 0.77, 0.95) lower odds of IADL disability. A similar association was found in the mixed-effects logistic regression models. During the follow-up period, 5,004 and 4,880 healthy participants developed ADL and IADL disabilities. Per 0.1-unit increase in baseline annual average Normalized Difference Vegetation Index (NDVI) was related to an OR of 0.95 of developing ADL disability (95% CI = 0.93, 0.98) and IADL disability (95% CI = 0.91, 0.98).

**Conclusions:**

Our study suggests that increasing green space is associated with lower odds of ADL and IADL disabilities, which may reduce caregiver burden of long-term care for Chinese older adults.

## Abstract 2

Supplemental Digital Content is available in the text.

**What this study adds**

More green space has been linked to better physical health. But there is no evidence on whether higher levels of residential greenness are associated with lower odds of activities of daily living (ADL) and instrumental activities of daily living (IADL) disabilities. To address this evidence gap, our study conducted cross-sectional and longitudinal analysis among 36,803 and 32,316 Chinese older adults, separately. We found that the highest quartile compared with the lowest quartile of residential greenness was associated with a 28% lower odds of ADL disability and 14% lower odds of IADL disability. Our study suggests that more green space might prevent or delay the onset of ADL and IADL disabilities, which has significant implications for reducing caregiver burden of long-term care for Chinese older adults.

## Introduction

Declines in vital capacity, muscular strength, and flexibility are more prevalent among older adults. These physiologic changes can cause assistance in performing activities of daily living (ADL) and instrumental activities of daily living (IADL).^1^ ADL consist of basic tasks in daily life: eating, bathing, dressing, toileting, transferring, and continence, reflecting essential self-care capacity.^2^ IADL indicate independent skills in a given environment, like shopping, cooking, and taking public transportation. ADL disability (unable to perform one or more of tasks) is more severe than IADL disability and often occurs at a more advanced age.^3^ Prior estimates of the prevalence of ADL disability among older adults 65 years of age or older varied regionally: 6.4%–18.6% in the United States, 26.6% in Spain, 1.6% in Hong Kong,^4–6^ which were less prevalent than IADL disability.^7^ ADL and IADL disabilities were linked to higher rates of mortality and comorbidities, more health service utilization, and long-term care.^8–10^ Furthermore, up to 20% of older adults with ADL disability reported unmet need for ADL assistance around the globe.^11–13^

A number of studies reported benefits of residential greenness on lower mortality rate.^14–16^ To our knowledge, there is no study on the effects of residential greenness on ADL and IADL disabilities among older adults. We hypothesized that higher levels of residential greenness were associated with lower odds of ADL and IADL disabilities and might prevent the onset of ADL and IADL disabilities. The potential mechanisms of health benefits of residential greenness on ADL and IADL could be that green space promotes physical activity and social engagement,^17,18^ which have been liked to better performance in ADL and IADL.^1,19–21^

China has the largest number of older population 65 years of age or older, about 148 million in 2017, which is currently under a rapid increase.^22^ A cross-sectional study in China showed that about 12.1%–16.8% of older adults 80 years of age or older needed assistance in ADL. However, more than half of them reported unmet needs for assistance in ADL, with higher vulnerabilities observed in rural areas.^23^ The prevalence of IADL disability was higher, up to 30.1% among older adults 65 years of age or older in Shanghai, China.^24^ We aimed to generate evidence on whether high levels of residential greenness could reduce odds of ADL and IADL disabilities among older adults, using the Chinese Longitudinal Healthy Longevity Survey (CLHLS).

## Methods

### Study population

Established in 1998, the CLHLS aimed to investigate the determinants of healthy longevity among Chinese older adults. The CLHLS used a multistage, stratified sampling design to recruit the participants from 631 randomly selected cities and counties, 22 out of 31 provinces in China. The sample sites represent about 85% of the Chinese population. This survey included only the participants 80 years of age or older in 1998 and 2000 and expanded to the participants 65 years of age or older since 2002. The CLHLS conducted follow-up surveys among the survivors and recruited new participants in 2000, 2002, 2005, 2008, 2011, 2014, and 2018. Extensive data on determinants of health have been collected, including demographic and socioeconomic characteristics, lifestyle, ADL, IADL, cognitive function, psychological status, and chronic diseases. Extra data regarding causes of death, health service utilization, and health status before death were collected from the family members of deceased participants. More details on study design and data quality could be found elsewhere.^25^

Our study utilized 2000, 2002, 2005, 2008, and 2011 waves of the CLHLS for the analysis of Normalized Difference Vegetation Index (NDVI) and ADL. The total sample size of five pooled waves consisted of 39,225 participants. We excluded the participants who were missing NDVI values (n = 353), were younger than 65 years (n = 545), and were missing particulate matter (PM)_2.5_ values (n = 1,524). We included 36,803 participants for the cross-sectional analysis. We additionally excluded those who were lost follow-up after the baseline survey (n = 5,766) and died before the follow-up survey (n = 11,961) for the longitudinal analysis (n = 19,076).

Because the variables used for coding IADL were available since 2002, we used 2002, 2005, 2008, and 2011 wave, including 34,342 participants, for the analysis of NDVI and IADL. We excluded the participants who were missing NDVI values (n = 17), were younger than 65 years (n = 545), and were missing PM_2.5_ values (n = 1,464). We included 32,316 participants for the cross-sectional analysis. We additionally excluded those who were lost follow-up after the baseline survey (n = 5,062) and died before the follow-up survey (n = 11,598) for the longitudinal analysis (n = 15,656).

### Greenness assessment

We calculated NDVI, a satellite image-based vegetation index, to reflect residential greenness. The plants absorb red visible light during the process of photosynthesis, while leaves reflect near-infrared light to scatter extra heat.^26^ NDVI is equal to the ratio of the difference between the near-infrared region and red visible reflectance to the sum of these two measures. NDVI ranges from −1.0 to 1.0, with larger values indicating more green space.^27,28^

Based on participants’ residential addresses, we obtained NDVI values from the Moderate-Resolution Imaging Spectro-Radiometer (MODIS) in the National Aeronautics and Space Administration’s Terra Satellite.^29,30^ Due to the temporal resolution of 16 days at MODIS, we measured two NDVI values for January, April, July, and October from 2000 to 2014 to reflect seasonal variation in greenness.

We calculated baseline NDVI in the 500 m radius around residential addresses of participants. Baseline NDVI was the annual average NDVI value at the year of study entry. In addition, we categorized baseline annual average NDVI into quartiles and calculated their 0.1 unit of values.

### ADL and IADL assessment

ADL assessed self-care capacity by using six self-reported questions: “Do you need assistance in bathing/dressing/toileting/transferring/eating/continence?”^31^ ADL had a continuous scale of zero to six with each question scored zero (without assistance) or one (with assistance). Higher ADL scores indicated more ADL disabilities. In our analysis, we dichotomized ADL scores to zero, defined as free of ADL disability, as the reference group, and one to six, defined as with ADL disability.

IADL assessed independent living skills including eight activities: visiting neighbors, shopping, cooking, washing clothes, walking 1 km, lifting 5 kg, crouching and standing up three times, and taking public transportation.^31^ We scored each activity zero (able to do without help) or one (need help). Ranging from zero to eight, we dichotomized IADL scores to zero, defined as free of IADL disability, as the reference group, and one to eight, defined as with IADL disability.

### Covariates

We measured a range of baseline characteristics, including age, sex, ethnicity, marital status, geographic region, urban/rural residence, education, occupation, financial support, social and leisure activity, smoking status, drinking status, physical activity, annual average PM_2.5_, and Mini-Mental State Examination (MMSE). We also generated a variable of time to reflect the number of years for each follow-up survey since entering the cohort for the longitudinal analysis.

Age was the difference between the interview dates and birth dates.^25^ We dichotomized sex to males and females. We divided ethnicity into Han Chinese and ethnic minorities (Hui, Korean, Manchurian, Mongolian, Yao, Zhuang, and others). We categorized marital status into married and not married at the time of interview (separated, or divorced, or widowed, or never married). We categorized the participants into seven geographical regions based on their residential addresses: Central China (Henan, Hubei, and Hunan Provinces), Eastern China (Anhui, Fujian, Jiangxi, Jiangsu, Shandong, Shanghai, and Zhejiang Provinces), Northeastern China (Heilongjiang, Jilin, and Liaoning Provinces), Northern China (Hebei, Shanxi, and Tianjin Provinces), Northwestern China (Shaanxi Province), Southern China (Guangdong, Guangxi, and Hainan Provinces), and Southwestern China (Chongqing and Sichuan Provinces). Residence was dichotomized to urban and rural area.

We divided education into groups of formal education (≥1-year schooling) and no formal education. We dichotomized occupation to professional work (professional and technical personnel, government and management) and nonprofessional work (agriculture, fishing, service, industry, and housework). We assessed financial support depending on whether participants relied on their own work and retirement wage (defined as financial independence) or received financial assistance from other family members (defined as financial dependence). We took into consideration seven activities into social and leisure activity index, including gardening, personal outdoor activities excluding exercise, raising poultry or pets, reading, playing cards or *mahjong*, listening to the radio or watching TV, and participating in organized social activities, with each scored zero or one.^32^ We evaluated smoking status by asking “smoke or not at present.” We evaluated drinking status and physical activity using similar questions. We used the adapted Chinese version of MMSE to reflect cognitive function. MMSE scores ranged from 0 to 30, with higher scores indicating better cognitive function. Based on participants’ residential addresses, we obtained the estimates of ground-level concentrations of PM_2.5_ from Atmospheric Composition Analysis Group.^33^ We used annual average PM_2.5_ at baseline year to indicate air pollution levels.

### Statistical analysis

We used binary logistic regression and mixed-effects logistic regression models to examine the associations between residential greenness and ADL disability, adjusted for age, sex, ethnicity, marital status, geographic region, urban/rural residence, education, occupation, financial support, social and leisure activity, smoking status, drinking status, physical activity, annual average PM_2.5_, and MMSE at baseline. Firstly, we used binary logistic regression models to assess the association between annual average NDVI and ADL disability at baseline. We stratified the analysis by age, sex, marital status, urban/rural residence, education, occupation, financial support, social and leisure activity index, smoking status, drinking status, and physical activity. Secondly, we applied mixed-effects logistic regression models to explore the relationship between annual average NDVI at baseline and ADL disability among the participants with follow-up surveys. Annual average NDVI was measured at baseline. The outcome ADL disability was repeatedly measured at baseline and subsequent follow-up surveys. The regression models were also adjusted for the variable of time elapsed. In addition, about half of the participants died or were lost before the follow-up survey. We conducted a sensitivity analysis on the participants with/without follow-up surveys using the binary logistic regression, to see how possible informative censoring may influence the association between residential greenness and ADL disability. Furthermore, we used binary logistic regression to test whether higher levels of annual average NDVI at baseline were related to lower odds of developing ADL disability among healthy participants who were free of ADL disability at baseline. We stratified the analysis by age group. We used the same statistical analysis to explore the association between NDVI and IADL disability.

We calculated odds ratios (ORs) and 95% confidence intervals (CIs) to estimate the magnitude of the associations. We reported the results of quartiles and per 0.1-unit increase in baseline annual average NDVI. We plotted the cubic splines with three knots to examine nonlinearity of the associations. We used STATA 14.0 (College Station, TX) for statistical analysis.

### Ethical approval

The CLHLS study was approved by the Institutional Review Board, Duke University (Pro00062871) and the Biomedical Ethics Committee, Peking University (IRB00001052-13074). All participants signed a written informed consent.

## Results

Table 1 describes the baseline characteristics of CLHLS participants. Participants (36,803) were included for the analysis of NDVI and ADL disabilities. The mean age was 88 years (SD = 11.5 years), 41.1% were male, and 76.1% lived in rural areas. The mean baseline NDVI was 0.40 (SD = 0.15). About 71.6% of participants were free of ADL disability at baseline.

**Table 1:**
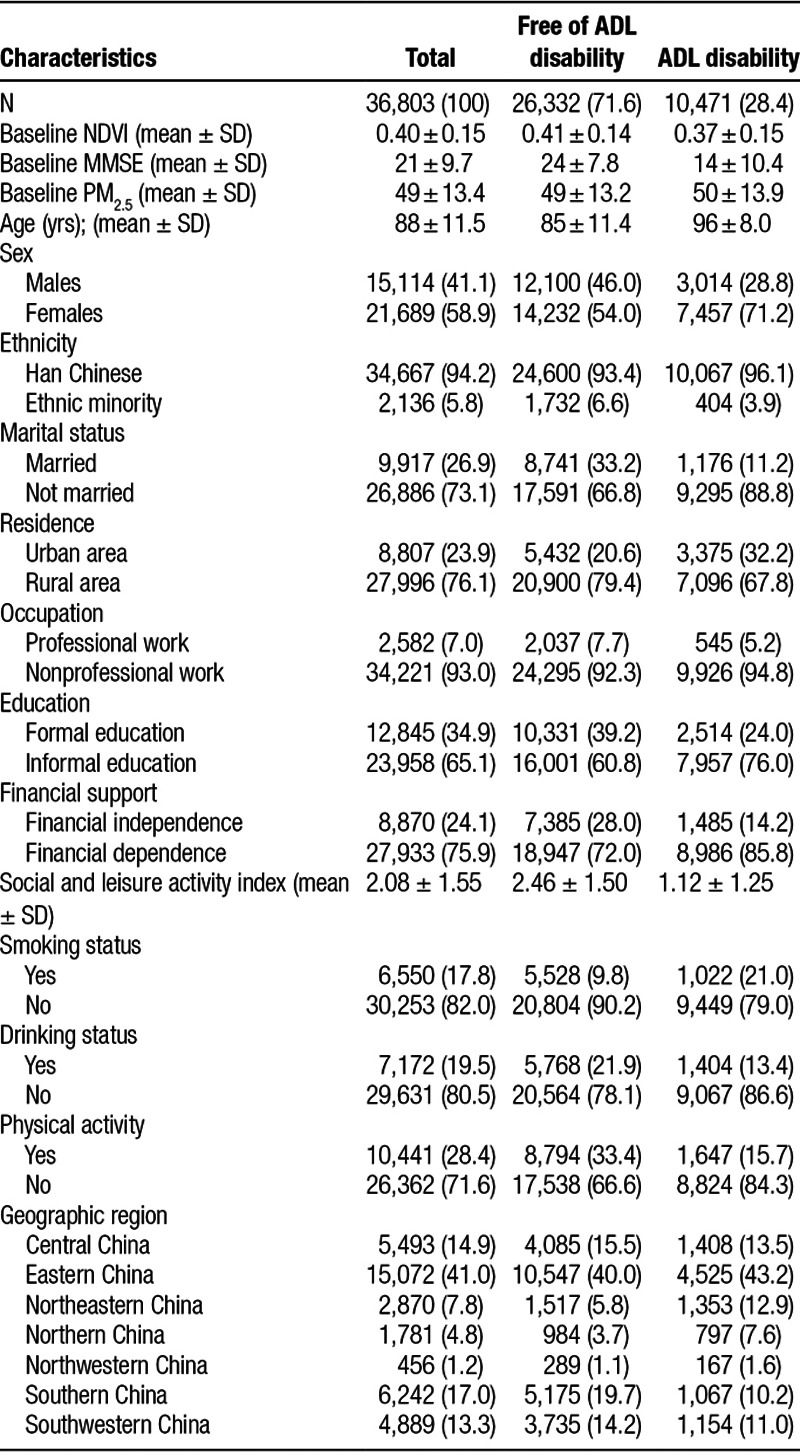
Baseline characteristics of CLHLS participants

**Figure 1.**
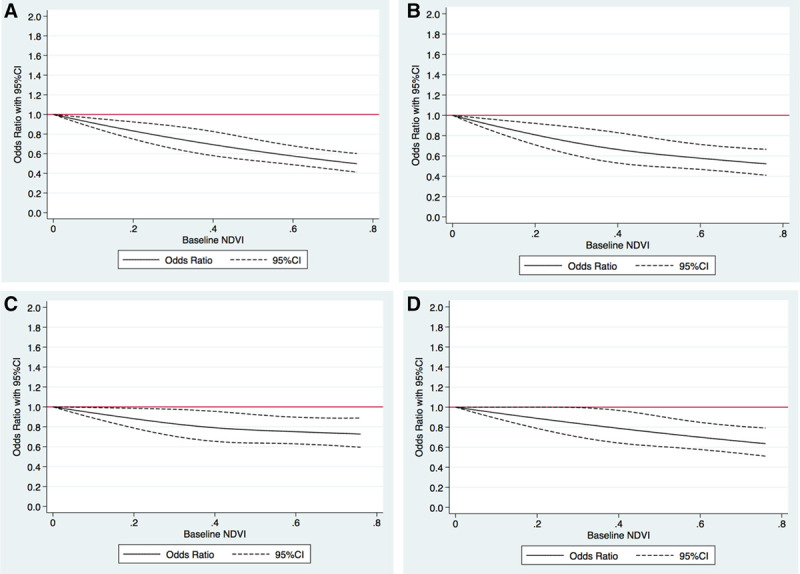
Curve association between baseline annual average NDVI, ADL, and IADL disabilities. A, The cross-sectional analysis between baseline annual average NDVI and ADL disabilities. B, The longitudinal analysis between baseline annual average NDVI and ADL disabilities. C, The cross-sectional analysis between baseline annual average NDVI and IADL disabilities. D, The longitudinal analysis between baseline annual average NDVI and IADL disabilities.

Table 2 shows ORs and 95% CIs for baseline annual average NDVI, ADL, and IADL disabilities. In the fully adjusted logistic regression, compared with the participants living in the lowest quartile of residential greenness, those in the highest quartile had a 28% (OR = 0.72; 95% CI = 0.65, 0.79) lower odds of ADL disability at baseline. Per 1-year increase in age was related to a 7% increase in ADL disability (OR = 1.07; 95% CI = 1.06, 1.07), indicating that living in the highest quartile of residential greenness has a protective effect size equivalent to roughly 4-year reduction in age, when comparing the ORs. Per 0.1-unit increase in baseline annual average NDVI was associated with an OR of 0.92 (95% CI = 0.90, 0.94) of ADL disability at baseline. The fully adjusted mixed-effects logistic regression showed the similar association (the highest quartile of NDVI: OR = 0.72; 95% CI = 0.64, 0.82; 0.1-unit of NDVI: OR = 0.92; 95% CI = 0.89, 0.95). Compare to those with follow-up surveys, the participants died or were lost before the follow-up survey were much older (92 vs. 84), had more ADL disability (41.3% vs. 16.5%), and lower MMSE scores (18 vs. 23) at baseline (Supplemental Table 1, http://links.lww.com/EE/A57). Our sensitivity analysis found a similar association between baseline annual average NDVI and ADL disabilities among those without follow-up surveys (0.1-unit NDVI: OR = 0.91; 95% CI = 0.88, 0.94). Cubic splines (Figure 1) also illustrate consistent findings with the cross-sectional analysis and longitudinal analysis, indicating a linear relationship between baseline annual average NDVI and ADL disabilities. Additionally, we observed similar protective effects of baseline annual average NDVI on IADL disability (the highest quartile of NDVI in the fully adjusted logistic regression: OR = 0.86, 95% CI = 0.77, 0.95; the highest quartile of NDVI in the fully adjusted mixed-effect logistic regression: OR = 0.83, 95% CI = 0.75, 0.93). The association between baseline annual average NDVI and IADL disabilities among those without follow-up surveys was weak (0.1-unit NDVI: OR = 0.96; 95% CI = 0.93, 1.00).

**Table 2:**
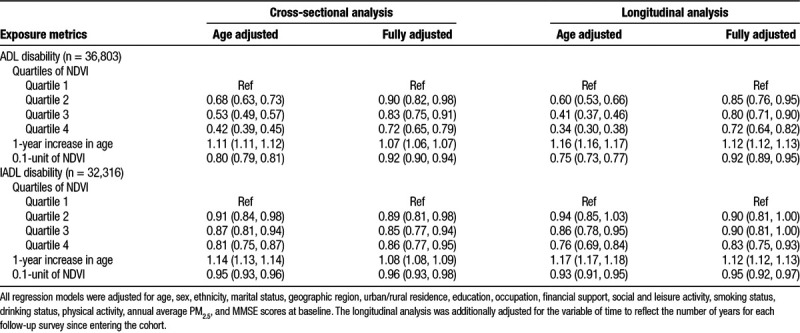
Odds ratios and 95% CI for baseline annual average NDVI, ADL, and IADL disabilities

**Figure 2.**
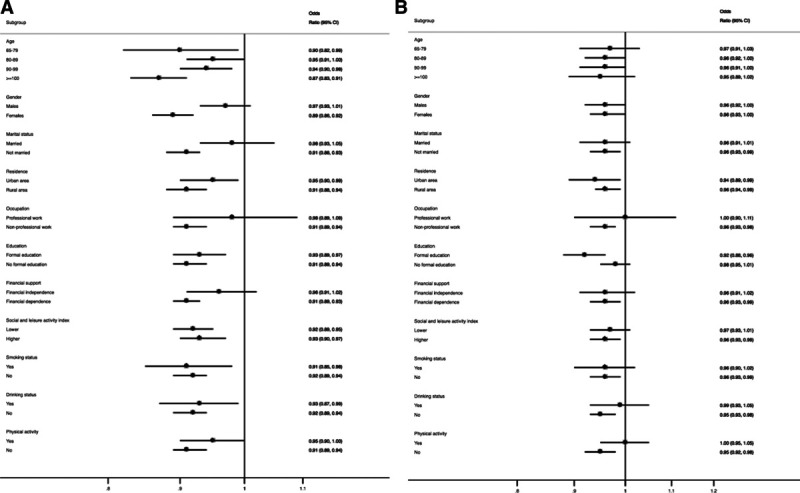
Stratified cross-sectional analysis for per 0.1-unit increase in baseline annual average NDVI, ADL, and IADL disabilities by subgroups. A, Stratified analysis on ADL disability. B, Stratified analysis on IADL disability. Note: All the stratified analysis were adjusted for age, sex, ethnicity, marital status, geographic region, urban/rural residence, education, occupation, financial support, social and leisure activity, smoking status, drinking status, physical activity, annual average PM_2.5_, and MMSE scores at baseline, except the stratified covariates.

Figure 2 reports the stratified analysis on per 0.1-unit increase in baseline annual average NDVI, ADL, and IADL disabilities. Slightly stronger protective effects on ADL disability were observed among the participants who were centenarians (100 years of age or older), females, not married, living in rural areas, had nonprofessional work, had no formal education, financial dependent, and not exercise. Additionally, the participants who lived in urban area, had formal education, no drink, and not exercise benefited more from residential greenness on IADL disability.

Table 3 presents ORs and 95% CI for per 0.1-unit increase in baseline annual average NDVI and odds of developing ADL and IADL disabilities among the healthy participants at baseline. During the follow-up surveys between 2000 and 2014, 5,004 out of 15,932 participants without ADL disability at baseline developed ADL disability. Per 0.1-unit increase in baseline annual average NDVI was related to an OR of 0.95 (95% CI = 0.92, 0.98) of developing ADL disability. The association was only statistically significant among the participants who were younger than 89 years old. In addition, from 2002 to 2014, 4,880 out of 9,904 participants without IADL disability at baseline developed IADL disability. Per 0.1-unit increase in baseline annual average NDVI was associated with a 5% (OR = 0.95; 95% CI = 0.91, 0.98) lower odds of developing IADL disability.

**Table 3:**
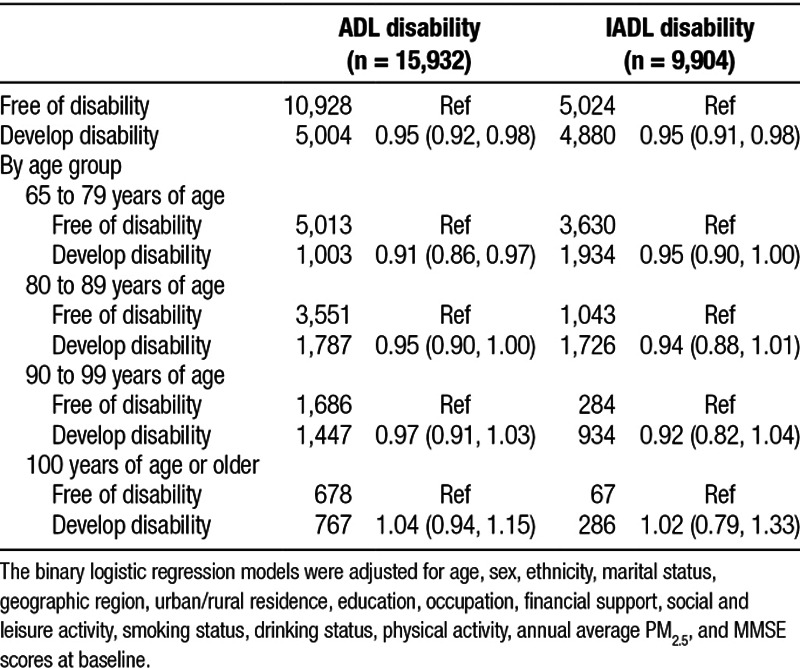
Odds ratios and 95% CI for per 0.1-unit increase in baseline annual average NDVI and development of ADL and IADL disabilities among healthy participants at baseline

## Discussion

Our study found that the highest quartile of residential greenness was associated with a 28% lower odds of ADL disability and a 17% lower odds of IADL disability compared with the lowest quartile. Both the cross-sectional and longitudinal analyses showed consistent findings. Together with our previous study,^34^ these findings make health benefits of residential greenness more convincing. Our study may also provide evidence on the potential mechanisms of residential greenness and mortality.

We observed that the more social and leisure activity and physical activity were associated with lower odds of ADL and IADL disabilities, which could be the potential mechanisms according to prior findings.^35–38^ Physical activity has been widely used as the key intervention on physical function. Studies have been showed that increased physical activity could prevent mobility disability, slow down mobility decline, and improve ADL and IADL functions among older adults.^1,39–41^ But some studies reported no associations.^42^ The inconsistent evidence is probably due to the different measurement of physical activity or lack of control for the confounding variables. Furthermore, social support is the other potential mechanism. Residential greenness could provide a supportive environment for more social engagement. Independent of physical activity, social activity is associated with slower ADL and IADL decline, although socially active people tend to be more physically active.^19,21,38,43,44^ However, the relationship could be reversed because physical function is essential for physical activity and social activity. Overall, the relationship among physical activity, social activity, ADL disability, and IADL disability is unclear. Additionally, our study lacked information on the usage pattern of green space around participants’ residence. It is hard to illustrate the mechanisms mediating the residential greenness and ADL disability.

Our stratified analysis showed that the participants who were not married, lived in rural areas, had no informal education, had nonprofessional work, and were financially dependent had stronger protective effects of residential greenness on ADL disability. One potential reason is the difference in residential greenness by subgroups. For instance, in our study, baseline annual average NDVI in rural areas is higher than in urban area (0.45 vs. 0.24). The difference in health effects could also be caused by the difference in time spent in green space, access to health care, baseline health status, and socioeconomic status. Health effects of green space differed by socioeconomic status in our study are in line with prior studies. An observational study of 40 million population in the United Kingdom reported that income-related health inequalities were smaller in more green areas.^45^ Other studies reported stronger protective effects of green space among people with lower socioeconomic status.^46–48^ However, the Swiss National Cohort showed stronger protective effects among people with higher income.^49^ In addition, we observed stronger protective effects on IADL disability among those who were living in urban areas and with formal education. The difference in the stratified analysis between ADL and IADL disabilities may be explained by differed health effects by socioeconomic status and different mechanisms of protective effects.

There are several limitations to our study. Firstly, although NDVI reflects overall greenness, it could not indicate exposure to a specific type of vegetation. We also had no information on activity patterns of participants. How these differences could influence the association between residential greenness and ADL and IADL disabilities is unclear. Secondly, there may be concerns in informative censoring in the longitudinal analysis. Our mixed-effects logistic regression models only included the participants with follow-up surveys, consist of only about half of the total sample. Those without follow-up surveys who were excluded from the longitudinal analysis were more vulnerable. However, our sensitivity analysis reported similar protective effects on ADL and IADL disabilities, compared with the longitudinal analysis. Thirdly, reverse causation relationship among residential greenness, ADL disability, and IADL disability is possible. In our study, the older adults with ADL or IADL disability live in the less green area (baseline annual average NDVI: 0.37 vs. 0.41) than those without ADL or IADL disability at baseline. This indicates that older adults with ADL disability probably may be more likely to live in urban areas, where has more healthcare resources but less green space. But our analysis of baseline annual average NDVI and development of ADL disability among the healthy participants indicates that the potential reverse association shall not greatly bias the association between residential greenness and ADL disability. Additionally, socioeconomic status is unlikely to confound the association between residential greenness and ADL disability. Our study found that the older adults living in greener area have lower socioeconomic status.

Our study has several strengths. To our knowledge, this is the first study assessing the effects of residential greenness on ADL and IADL disabilities among older adults. The protective effects of residential greenness observed in our study add to the mechanism of residential greenness and overall mortality and also provide evidence for preventing ADL and IADL disabilities in further research. In addition, our study included a large size of nationally representative sample, from 22 provinces in Mainland, China. Furthermore, our study used both the cross-sectional and longitudinal designs, which showed consistent findings. We used aerial satellite-derived measurements, which take into consideration the seasonal variation of greenness, for the exposure assessment. This more objective measurement of greenness exposure by using NDVI and mixed analysis provided stronger evidence on the relationship among residential greenness, ADL disability, and IADL disability.

## Conclusions

Our study showed that higher levels of residential greenness were associated with lower odds of ADL and IADL disabilities among older adults. These protective effects indicated that more green space might help prevent or delay the onset of ADL and IADL disabilities. Our findings have public health implications for reducing caregiver burden of long-term care for the large and ongoing increase group of Chinese older adults.

## Acknowledgments

We would like to acknowledge Chen Bai, Chengcheng Qiu, Anran Tan, Jingyu Tong, and Longkai Zang for their contribution in geocoding residential addresses; Yinq-Rong Chern for NDVI data preparation.

## Conflicts of interest statement

The authors declare that they have no conflicts of interest with regard to the content of this report.

This study was supported by Bill & Melinda Gates Foundation (Grant Number: OOP1148464), and NCI (Grant Number: R00 CA201542). The data analyzed in this paper were provided by the Chinese Longitudinal Healthy Longevity Study (CLHLS) which has been jointly supported by National Natural Sciences Foundation of China (71490732) and the National Key R&D Program of China (2018YFC2000400).

Data used in this study are available upon request from Center for Healthy Aging and Development Studies, National School of Development, Peking University. Computer code will be made available upon request.

## Supplementary Material
