# Supplementary material for: Residential greenness, activities of daily living, and instrumental activities of daily living: A longitudinal cohort study of older adults in China
Source: Environ Epidemiol. 2019 Aug 30;3(5):e065. doi: 10.1097/EE9.0000000000000065 (PMC7608893; doi:10.1097/EE9.0000000000000065)
Supplement: Supplementary file 1 [file ee9-3-e065-s001.pdf]

Supplementary Table 1. Baseline annual average NDVI, ADL and IADL for the participants with/without follow-up surveys

	All participants	Participants with follow-up surveys	Participants without follow-up surveys		
			All	Death	Lost follow-up
ADL					
N	36,803	19,076	17,727	11,961	5,766
Baseline NDVI (mean±SD)	0.40±0.15	0.41±0.14	0.39±0.15	0.41±0.14	0.35±0.16
Baseline ADL (n, %)					
Free of ADL disability	26,332 (71.6)	15,922 (83.5)	10,410 (58.7)	6,499 (54.3)	3,911 (67.8)
ADL disability	10,471 (28.4)	3,154 (16.5)	7,317 (41.3)	5,462 (45.7)	1,855 (32.2)
Baseline MMSE score (mean±SD)	21±9.7	23±7.9	18±10.6	16±10.7	21±9.7
Age (mean±SD)	88±11.5	84±11.4	92±10.2	94±8.6	88±11.8
0.1-unit of NDVI*	0.92 (0.90, 0.94)	0.92 (0.89, 0.95)	0.91 (0.88, 0.94)	0.90 (0.87, 0.94)	0.91 (0.86, 0.97)
IADL					
N	32,316	15,656	16,660	11,598	5,062
Baseline NDVI (mean±SD)	0.39±0.15	0.40±0.14	0.38±0.15	0.40±0.14	0.35±0.16
Baseline IADL (n, %)					
Free of IADL disability	15,273 (47.3)	9,904 (62.3)	5,369 (32.2)	2,929 (25.2)	2,440 (48.2)
IADL disability	17,043 (52.7)	5,752 (36.7)	11,291 (67.8)	8,669 (74.8)	2,622 (51.8)
Baseline MMSE score (mean±SD)	20±9.8	23±7.9	17±10.5	16±10.4	21±9.7
Age (mean±SD)	88±11.9	83±11.6	92±10.3	94±8.6	87±12.2
0.1-unit of NDVI*	0.96 (0.93, 0.98)	0.95 (0.92, 0.97)	0.96 (0.93, 1.00)	0.95 (0.91, 0.99)	0.98 (0.92, 1.04)

Note: All the regression models were adjusted for age, gender, ethnicity, marital status, geographic region, urban/rural residence, education, occupation, financial support, social and leisure activity, smoking status, drinking status, physical activity, annual average $PM_{2.5}$, and MMSE scores at baseline.
